# Genome-wide analysis of the homeodomain-leucine zipper family in *Lotus japonicus* and the overexpression of *LjHDZ7* in Arabidopsis for salt tolerance

**DOI:** 10.3389/fpls.2022.955199

**Published:** 2022-09-14

**Authors:** Dan Wang, Yuan Gong, Yang Li, Shuming Nie

**Affiliations:** Key Laboratory of Southwest China Wildlife Resources Conservation (Ministry of Education), College of Life Science, China West Normal University, Nanchong, China

**Keywords:** HD-ZIP, *Lotus japonicus*, gene expression, *LjHDZ7*, salt stress

## Abstract

The homeodomain-leucine zipper (HD-Zip) family participates in plant growth, development, and stress responses. Here, 40 HD-Zip transcription factors of *Lotus japonicus* were identified and gave an overview of the phylogeny and gene structures. The expression pattern of these candidate genes was determined in different organs and their response to abiotic stresses, including cold, heat, polyethylene glycol and salinity. The expression of the *LjHDZ7* was strongly induced by abiotic stress, especially salt stress. Subsequently, *LjHDZ7* gene was overexpressed in Arabidopsis. The transgenic plants grew obviously better than Col-0 plants under salt stress. Furthermore, *LjHDZ7* transgenic lines accumulated higher proline contents and showed lower electrolyte leakage and MDA contents than Col-0 plants under salt stress. Antioxidant activities of the *LjHDZ7* overexpression lines leaf were significantly higher than those of the Col-0 plants under salt stress. The concentration of Na^+^ ion in *LjHDZ7* overexpression lines was significantly lower than that of Col-0 in leaf and root parts. The concentration of K^+^ ion in *LjHDZ7* overexpression lines was significantly higher than that of Col-0 in the leaf parts. Therefore, these results showed that overexpression of *LjHDZ7* increased resistance to salt stress in transgenic Arabidopsis plants, and certain genes of this family can be used as valuable tools for improving abiotic stresses.

## Introduction

Homeodomain-leucine zipper (HD-Zip) contains a conserved homology domain (HD) motif and a leucine zipper (LZ) motif (Ariel et al., 2007). HD-Zip is a unique transcription factor (TF) that is present in a variety of plants (Henriksson et al., 2005; Sakuma et al., 2013). HD-Zip is involved in various regulatory processes of plant growth and development and responses to various external environmental signals (Wang et al., 2013; Ribone et al., 2015). HD-Zip is expressed in various tissues and organs of plants and plays an important role in different growth stages (Brant et al., 2014). Excluding the conserved HD-Zip domain, these family members have different amino acid sequence lengths and protein structures (Arce et al., 2011).

The HD-Zip gene family can be divided into four subfamilies, I to IV, according to the amino acid sequence (Capella et al., 2015). Each subfamily member has a unique function and forms a complex interaction network. HD-Zip I positively responds to various environmental stresses, such as drought, salty, pathogens, chilling injury, light signal response and hormone response (Romani et al., 2016). The expression of HD-Zip II genes is affected by light. The TFs of this family play important roles in the development of plant organs, shade avoidance response and hormone response (Turchi et al., 2015; Carabelli et al., 2018; He et al., 2021). HD-Zip III has obvious effects on the development of plant embryos, the formation of microtubule tissue, the differentiation of apical provinces and the polar transport of auxin (Yang S. et al., 2018). HD-Zip IV promotes the differentiation of epidermal cells in plant organs and the formation of anthocyanins (Nakamura et al., 2006; Zalewski et al., 2013).

Plants often face various biological and abiotic stresses during growth and development. Under various stresses, plants form a complex signal transduction network to resist adversity. The HD-Zip gene family has been reported to participate in adversity stress in many species (Zhang J. et al., 2020). HD-Zip genes play a crucial role in plant protection against pathogens and abiotic stresses (Gao et al., 2015). Overexpression of the wheat TaHDZipI-5 gene improved transgenic wheat freezing and drought resistance (Yang et al., 2017). Overexpression of PsnHDZ63 could increase reactive oxygen species scavenging ability and enhanced salt stress tolerance in transgenic *Populus simonii* × *P. nigra* (Guo et al., 2021). Overexpression of the rice HD-Zip I gene *OsHOX24* imparted higher sensitivity to stress hormones, ABA, and abiotic stresses in transgenic Arabidopsis plants than in wild-type plants (Annapurna et al., 2016; Tang et al., 2019). These studies showed that the HD-Zip gene family could play an important role in plant resistance to abiotic stress.

Homeodomain-leucine zipper genes from model plants to higher plants have been identified and analyzed. For example, the model plants *Arabidopsis thaliana* (Nakamura et al., 2006), rice (*Oryza sativa* L.) (Zhang et al., 2012), maize (*Zea mays* L., Gramineae family), moso bamboo (*Phyllostachys edulis*) (Gao et al., 2021), potato (*Solanum tuberosum* L., Solanaceae family) (Li et al., 2019), and pear (*Pyrus pyrifolia*, rose family) (Yang Q. et al., 2018). Many families and genera of the HD-Zip gene family in these species have been identified and analyzed (Yang S. et al., 2022). The functions of a number of HD-Zip genes have been studied (Zhao et al., 2014), and the regulatory mechanisms of certain HD-Zip genes have been clarified (Zhang et al., 2012). However, the evolution of these genes in specific species and groups remains elusive; moreover, limited information is available on the HD-Zip family in *Lotus japonicus*.

*Lotus japonicus* is an important leguminous forage that is used as a protein feed source, biological nitrogen fixation resource and ecological conservation species. In this study, we identified 40 HD-Zip TFs of *Lotus japonicus*. To provide insights on HD-Zip gene evolution and function, we performed analyses of the phylogeny, proteins, gene structure, promoter *cis*-elements, and tissue and stress expression patterns. We found that the expression of *LjHDZ7* was strongly induced by salt stress. Further studies showed that overexpression of *LjHDZ7* increased the salt resistance of transgenic Arabidopsis plants. Our results will be helpful in understanding the evolutionary relationships, proteins, gene structures, and biological functions of HD-Zip TFs in *Lotus japonicus* and will provide a foundation for further elucidating the salt resistance mechanism of the *LjHDZ7* gene.

## Materials and methods

### Phylogenetic and physicochemical characteristics of the homeodomain-leucine zipper protein sequence

The protein sequences of the *Lotus japonicus*, *Medicago truncatula*, *Trifolium pretense*, and *Arabidopsis thaliana* HD-Zip family were downloaded from the plant TFDB V4.0 database^1^. The candidate protein sequences were screened and identified using the CDD^2^ and SMART^3^, and manual elimination was performed to remove redundant sequences and sequences without typical domains. The HD-Zip family was finally determined by the protein sequence of the conserved domain.

To investigate the phylogenetic relationships of the HD-Zip members among different plants, multiple HD-Zip amino acid sequences were aligned via Clustal 2.1 software using the default settings to examine the evolutionary relationships among the sequences. A phylogenetic tree was constructed with MEGA7.0 software with 1000 bootstrap replicates according to the maximum-likelihood (ML) method (Li et al., 2022).

The *Lotus japonicus* HD-Zip protein structural characteristics, isoelectric point (pI), molecular weight (MW) and Grand average of hydropathicity (GRAVY) values were determined using the ExPASy ProtParam tool^4^. The subcellular localization of HD-Zip genes was predicted using the online tools UniProt^5^ and WoLF PSORT^6^.

### Conserved motif and domain analysis

Conserved protein motifs of the HD-Zip family of *Lotus japonicus* were predicted using MEME Suite^7^ with the default settings. The details of the top 10 predicted motifs were obtained from the MEME suite. The conserved domains of the HD-Zip family of *Lotus japonicus* were predicted using NCBI-CDDCDD (see text footnote 2). The distribution of the conserved domain and motif were drawn via the visualization tool in TBtools software. The three-dimensional structure (3D) of LjHD-Zip proteins was predicted in the Phyre2^8^ using the default advanced settings.

### Gene structure and chromosome localization

The DNA sequences of the HD-Zip gene family of *Lotus japonicus* were obtained from an online transcriptome and genome database^9^. These gene structures were displayed by server GSDS2.0^10^, and the intron/exon distribution pattern of each LjHD-Zip is illustrated. These gene chromosome localizations were illustrated using MG2C_V2.1^11^.

### Promoter sequence analysis

The 2000 bp upstream sequence of the initiation codon of each LjHD-Zip gene was extracted from the corresponding scaffolds (see text footnote 10). Then, the cis-elements in the promoters of each LjHD-Zip gene were predicted using the PlantCARE server^12^. The distribution of the cis-elements in the promoter was drawn via the visualization tool for *cis*-elements in TBtools software.

### Plant growth conditions and stress treatments

*Lotus japonicus* ecotype “MG20” was used in this study. We selected seeds with full grains, planted them in pots with perlite, grew them in a controlled environment with a photoperiod of 16 h/8 h (light/dark) at 24°C and 70% relative humidity in a growth chamber for 60 days, and then started the treatment. For the drought treatment, we removed the original culture solution and then watered the solution with 15% polyethylene glycol (PEG) to simulate drought stress. The leaf sample material was measured at 0, 3, 6, 12, and 24 h. For the salt treatment, the original culture solution was removed and the solution was watered with 250 mM NaCl. The leaf sample material was measured at 0, 3, 6, 12, and 24 h. For the low-temperature treatment, the seedlings were transported to an incubator at 4°C and the leaf sample material was measured at 0, 3, 6, 12, and 24 h. For the high-temperature treatment, the seedlings were transported to an incubator at 42°C and the leaf sample material was measured at 0, 3, 6, 12, and 24 h. Each treatment included three replicates. Each replicate included 10 plants.

Seed germination in the salt stress treatment was evaluated by cultivating seeds of the transgenic Arabidopsis line and Col-0 were grown on MS medium containing 150 mM NaCl for 7 days. Leaf growth and root length were assessed by cultivating the seeds of Col-0 and overexpression *LjHDZ7* transgenic Arabidopsis in MS medium for 7 days, transferring the seedlings to vertical plate culture with MS medium containing 150 mM NaCl, and then cultivating the seedlings for 10 days before investigation. For the salt stress treatments, seeds of Col-0 and overexpression *LjHDZ7* transgenic Arabidopsis were grown in MS medium for 7 days, transplanted into nutrient soil, grown for 35 days, and watered with 300 mM NaCl. Col-0 and transgenic Arabidopsis shoot were harvested and oven dried at 80°C for 24 h to obtain dry weights (DW) (Wu et al., 2018).

Total RNA was extracted from leaves using plant isolation kits (Sangon Biotech, Shanghai, China, Cat. #B518631). cDNA (complementary DNA) was prepared from total RNA with random primers using cDNA synthesis kits (Vazyme, Nanjing, China, Cat. #R312-02) (Nie et al., 2022). The resulting cDNA was used as a template for qPCR (quantitative real-time PCR) analysis using SYBR Green real-time PCR master mix (Vazyme). The qPCR assays were performed in triplicate using a real-time PCR system (Bio-Rad, Hercules, CA, United States) based on the manufacturer’s instructions (Wang et al., 2022b). The primers used for the qRT–PCR sets are listed in Supplementary Table 7. *Lotus japonicus* LjUbi (*Lotus* ubiquitin gene) was used as a reference gene, and then expression analyses with different treatment times were performed to determine the gene expression under different stress treatments, with 0 h used as a reference. The relative gene expression was quantified using the 2^–△^^△^^CT^ method (Livak and Schmittgen, 2001). The PCR program was as follows: 1 cycle of 95°C for 30 s, followed by 40 cycles of 95°C for 10 s, 60°C for 30 s, and 72° for 30 s (Wang et al., 2022a). The resulting clusters were visualized with MeV software.

### Interacting protein predictions

The protein interaction network was analyzed and predicted using STRING software^13^, using *Arabidopsis* as the background and the default advanced settings. Functional enrichment analysis was performed using STRING.

### Electrolyte leakage

Electrolyte leakage was determined by using the conductivity meter method (Su et al., 2015). The leaves were cut into pieces and placed in deionized water in the dark for 12 h, and then the electrical conductivity (EC1) was measured. The sample leaves were boiled for 30 min in water, and after cooling to room temperature, the electrical conductivity (EC2) was measured. Leakage was calculated by the formula:

Relative electrolyte leakage (%) = EC1/EC2 × 100%, where EC1 and EC2 refer to electric conductivity of live leaves and boiled leaves, respectively.

### Malondialdehyde content

The malondialdehyde (MDA) contents were determined using the thiobarbituric acid reaction method (He et al., 2018). Fresh leaf samples were powdered in liquid nitrogen and homogenized with trichloroacetic acid (TCA). The supernatant was centrifuged and extracted and then mixed with thiobarbituric acid (TBA). The mixture was boiled and centrifuged, and then the absorbance was measured at wavelengths of 450, 532, and 600 nm. The contents were calculated by the formula:

Malondialdehyde concentration (C) (μmol/L) = 6.45 × (OD532 nm – OD600 nm) – 0.56 OD450 nm. OD450, OD532, and OD600 represented the absorbance value at 450, 532, and 600 nm, respectively. MDA content (μmol/g) = CV/1000 W. C: MDA concentration; V, volume extract; W, fresh weight.

### Proline content

The proline content was determined using the acidic ninhydrin reaction method (Bai et al., 2017). Fresh leaf samples were powdered in liquid nitrogen, homogenized in sulfosalicylic acid and heated in boiling water. The samples were centrifuged, and the supernatant was mixed with acidic ninhydrin and glacial acetic acid. After cooling, the samples were mixed with toluene, the supernatant was collected, and then the absorbance was determined at 520 nm. The content was calculated by the formula:

Proline contents (μg/g) = (C × VT/VS)/W. C: The standard curve for absorbance value at 520 nm (A520), A520 = 0.0512C + 0.001, *R*^2^ = 0.9955; VT, volume extract; VS, supernatant volume; W, fresh weight.

### Antioxidant enzyme activities

Superoxide dismutase (SOD), peroxidase (POD) and Catalase (CAT) activity was determined following the protocols proposed by the corresponding assay kit (Geruisi Biotechnology, Suzhou, China), respectively. The homogenate mixed with 0.1 g fresh sample and 1 mL extraction solution was centrifuged at 8,000 *g* for 10 min at 4°C, after which the supernatant was used to assay the activity of antioxidant enzymes. One unit of SOD was defined as the amount of enzyme that caused 50% of NBT photochemical reduction at 450 nm. One unit of POD was the change of 0.01 in a 1 mL reaction mixture at 470 nm per minute and per 1 g tissue. One unit of CAT was the 1 nmol H_2_O_2_ degradation at 510 nm per minute and per 1 mg tissue (Dong et al., 2021; Lü et al., 2022; Zhang et al., 2022).

### Measurements of Na^+^ and K^+^ concentration

The leaves and roots from Col-0 and *LjHDZ7* overexpression plants were separately harvested, dried for 48 h at 80°C and then ground to powder. The same mass leaves and roots powder was extracted in concentrated (69%, v/v) HNO_3_ for 24 h at room temperature (Li et al., 2016). Na^+^ and K^+^ concentrations were determined by using a flame photometer (Sherwood flame photometer-410, Cambridge, United Kingdom).

### Statistical analysis

The data in this paper were analyzed using SPSS version 17.0 and the least significant difference (LSD) test. The three biological replicates were performed. Mean and standard error values were calculated for the variable comparisons. Values of *p* < 0.05 were considered statistically significant.

## Results

### Phylogenetic relationship and classification of the homeodomain-leucine zipper family

**FIGURE 1:**
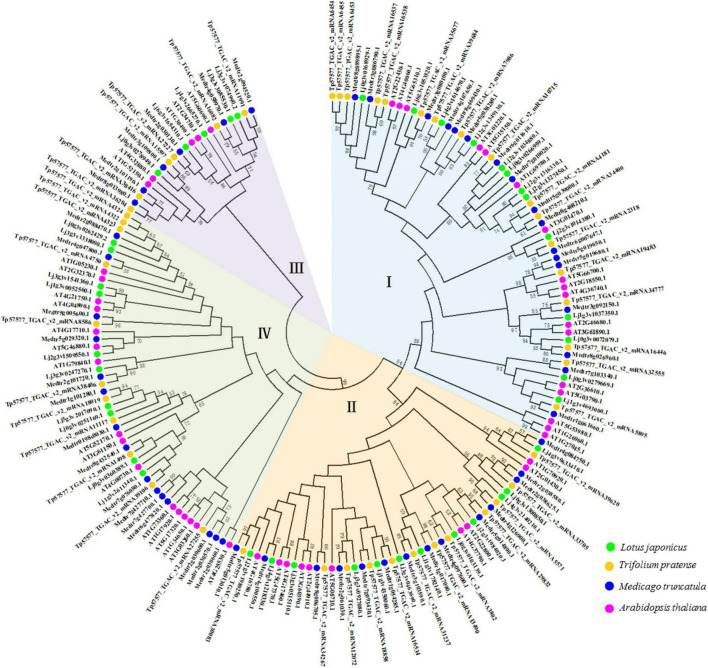
Phylogenetic tree of the HD-Zip proteins of three leguminous forage plants and *Arabidopsis thaliana*. Relationship among HD-Zip proteins constructed using the maximum-likelihood method. Different colored branches (blue, yellow, purple, and green) represent different groups (I, II, III, and IV). Labels above the nodes represent HD-Zip proteins from the source species.

A phylogenetic tree was constructed using full-length protein sequences obtained for *Lotus japonicus*, *Medicago truncatula*, *Trifolium pretense*, and *Arabidopsis thaliana*. HD-Zip genes were divided into four subfamilies (Figure 1). The number of HD-Zip genes in subgroup I was the largest (Supplementary Figure 1A), and the subfamily genes accounted for 35.42, 32.69, 36.96, and 32.50% of the whole family in *Arabidopsis thaliana*, *Medicago truncatula*, *Trifolium pretense*, and *Lotus japonicus*, respectively (Supplementary Figure 2). Longer amino acid sequences were obtained for HD-Zip III and HD-Zip IV, while shorter sequences were obtained for HD-Zip I and HD-Zip II (Supplementary Figures 1B–E).

The physicochemical properties of the amino acids encoded in the three leguminous forage species were analyzed (Supplementary Tables 1–4). The largest and smallest proteins were Tp57577_TGAC_v2_mRNA16082 of the HD-Zip III subfamily and Lj4g3v0633410.1 of the HD-Zip II subfamily, respectively, which had peptide chain lengths of 896 aa and 117 aa, respectively. The pI of 70.07% of the HD-Zip family proteins was less than 7, and they were weakly acidic. The pI ranged from 4.44 to 9.80, and the span was very large, indicating that the subfamily members came from different physiological environments. The physicochemical characteristics of *Lotus japonicus* were further predicted. The total average hydropathicity GRAVY values were negative and ranged from –1.186 to –0.116, thus implying their hydrophilic nature (Supplementary Table 1). The subcellular localization of *Lotus japonicus* HD-Zip proteins was in the nucleus, as indicated by UniProt software and WoLF PSORT (Supplementary Tables 1, 5).

### Conservative domain analysis of homeodomain-leucine zipper in *Lotus japonicus*

The structure of HD-Zip proteins was analyzed in *Lotus japonicus* (Supplementary Figure 3). The multiple sequence alignment showed that HD-Zip I and HD-Zip II have higher sequence similarity and HD-Zip III and HD-Zip IV have a closer homologous evolution relationship. We examined the LjHD-Zip proteins for discovery motifs using the MEME program, and the top 10 enriched motifs were identified (Supplementary Table 6). The structural characteristics of the motif are shown in Supplementary Figure 4. From the conservative domain and motif analysis of LjHD-Zip proteins, HD-Zip I and HD-Zip II had the same conservative domain while HD-Zip III and HD-Zip IV had more similar structures. The advanced structure of plant proteins is related to their biological function and activity. The three-dimensional (3D) structure of LjHD-Zip proteins was studied by modeling predictions (Supplementary Figure 5). HD-Zip I and HD-Zip II were highly similar in their α-helix and β-sheet structures, while HD-Zip III and HD-Zip IV had complex protein structures.

### Gene structure of homeodomain-leucine zipper in *Lotus japonicus*

We analyzed and drew the exon and intron structure diagram of HD-Zip genes in *Lotus japonicus* (Supplementary Figure 6A). All LjHD-Zip gene members have introns, and the number of exons in subgroup I and II ranged from 2 to 4, those in subgroup III ranged from 12 to 18 except for one, and those in subgroup IV ranged from 7 to 11 (Supplementary Figure 6B). The number of introns in each subgroup was relatively stable, and the gene structure of subgroup III and IV was relatively complex.

All 40 LjHD-Zip gene members were investigated for chromosome localization. They were unevenly distributed on 6 chromosomes (Supplementary Figure 7A), and the genes of each subgroup were unevenly distributed on each chromosome (Supplementary Figure 7B). The unbalanced distribution of HD-Zip genes on the chromosomes was related to the functional characteristics of the genes over the process of evolution.

### Promoter regulatory element of homeodomain-leucine zipper in *Lotus japonicus*

**FIGURE 2:**
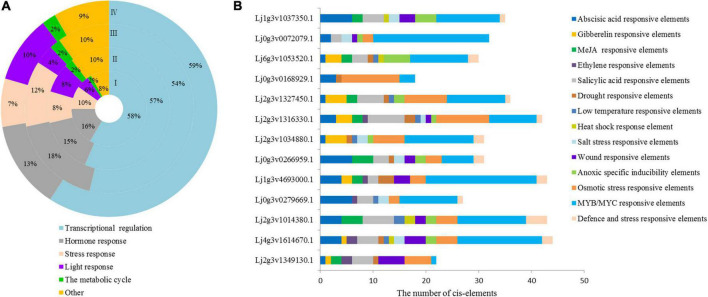
*Cis*-element statistical analysis of HD-Zip promoters of *Lotus japonicus*. **(A)** Different *cis*-element statistical analysis of four subfamilies. **(B)** *Cis*-element statistical analysis of HD-Zip I family gene promoters related to stress and hormone responses. Different *cis*-elements with similar functions are shown in the same color.

The 2000 bp upstream sequence of the LjHD-Zip gene was intercepted, and the regulatory elements of each gene promoter were obtained by PlantCARE (Supplementary Figure 8). The promoter region of the HD-Zip genes had a number of elements involved in different metabolic regulatory elements, and a total of 95 *cis*-elements were detected in *Lotus japonicus*. We classified these elements into 6 categories and analyzed the regulatory elements of each subgroup. All identified cis-elements had stress and hormone response elements, which accounted for 10% and 16% of *Lotus japonicus* HD-Zip I, respectively (Figure 2A). The 13-gene promoter of HD-Zip I contained 14 types of stress-related cis-elements, including ABA, GA, MeJA, ETH, SA, drought, temperature, and salt stress cis-elements (Figure 2B). The Lj1g3v1037350.1, Lj0g3v0266959.1, Lj2g3v1034880.1, Lj2g3v1316330.1, Lj2g3v1327450.1, and Lj0g3v0072079.1 of HD-Zip I were selected for intensive study according to the ABA and salt stress-related *cis*-elements. We further analyzed the regulatory elements of each gene promoter of HD-ZIP II, III, and IV all genes (Supplementary Figure 9). Three genes of each HD-Zip family were selected for analysis expression level under different stress types and stress time points.

### Gene expression patterns of different tissues and responses to abiotic stress

**FIGURE 3:**
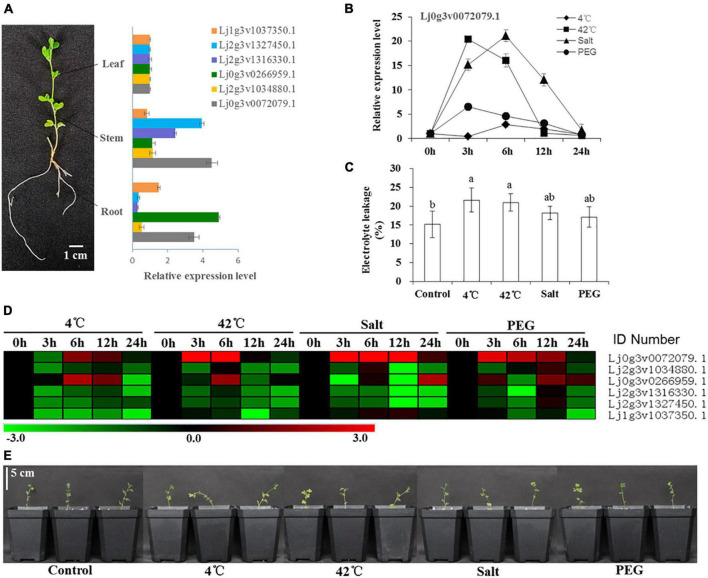
Expression levels of six HD-Zip I genes under various stresses in different times. **(A)** Tissue specificity analysis of six HD-Zip I genes and mRNA expression levels in leaves, stems and roots of *Lotus japonicus*. **(B)** Expression level of Lj0g3v0072079.1 during different stresses and times. **(C)** Electrolyte leakage of *Lotus japonicus* under different stresses at 24 h. **(D)** Heatmap of six HD-Zip I genes under 4°C, 42°C, salt and PEG stresses at different times. **(E)** Phenotypes of *Lotus japonicus* seedlings under different stresses at 24 h. Data values are the means ± SD of three independent biological samples. Different letters indicate significance using the LSD test (*p* < 0.05).

The expression of 6 HD-Zip I genes in different tissues (root, stem, leaf) was analyzed by qRT–PCR (Figure 3A). The relative expression of Lj2g3v1327450.1, Lj2g3v1316330.1, and Lj0g3v0072079.1 was higher than that of the other three genes in the stems. The expression of Lj1g3v1037350.1, Lj0g3v0266959.1, and Lj0g3v0072079.1 was higher than that of the other genes in the roots. After stress treatment for 24 h, these seedlings showed different degrees of wilting and damage (Figure 3E). The electrolyte leakage of the 4 and 42°C treatment leaves was significantly higher than that of the control leaves (Figure 3C). Our results showed that these six genes had differential expression abundance under different stress types and stress time points (Figure 3D). Similarly, nine genes of HD-ZIP II, III, and IV had also differential expression abundance under different stress types and stress time points (Supplementary Figure 10). The expression of Lj0g3v0072079.1 was strongly induced by the stress treatments, especially salt stress. The expression of Lj0g3v0072079.1 rapidly increased and then decreased after the 42°C and PEG treatments for 3 h, while it decreased after the 4°C and salt treatments for 6 h (Figure 3B). Therefore, Lj0g3v0072079.1 may play an important role in stress responses.

### Interacting protein predictions

The interacting proteins of Lj0g3v0072079.1 were predicted (Supplementary Figure 11A). The red node query protein was interacted with the stress response proteins RD26 and WRKY and ABA signaling-related proteins ABI (ABA-insensitive), HAB (hypersensitive to ABA), PYL (regulatory component of ABA receptors) and PP2C (protein phosphatase 2C). We further predicted the interaction protein networks of Lj0g3v0072079.1, with *Medicago truncatula* as the background, and obtained stress-related proteins (Supplementary Figure 12). We performed a Gene Ontology analysis and found that the functional enrichment network of Lj0g3v0072079.1 proteins participated in the ABA-activated signaling pathway (Supplementary Table 8). The *Lotus japonicus* PP2C gene families Lj4g3v3044980.2 and Lj6g3v1211810.1 were found through homology comparisons (Supplementary Figure 13). The degrees of up- or down-regulation of Lj4g3v3044980.2 and Lj6g3v1211810.1 were different under different stresses (Supplementary Figure 11B). These results show that Lj0g3v0072079.1 may be involved in stress responses by regulating the ABA signaling pathway.

### Overexpression of *LjHDZ7* improves salt tolerance in transgenic Arabidopsis

**FIGURE 4:**
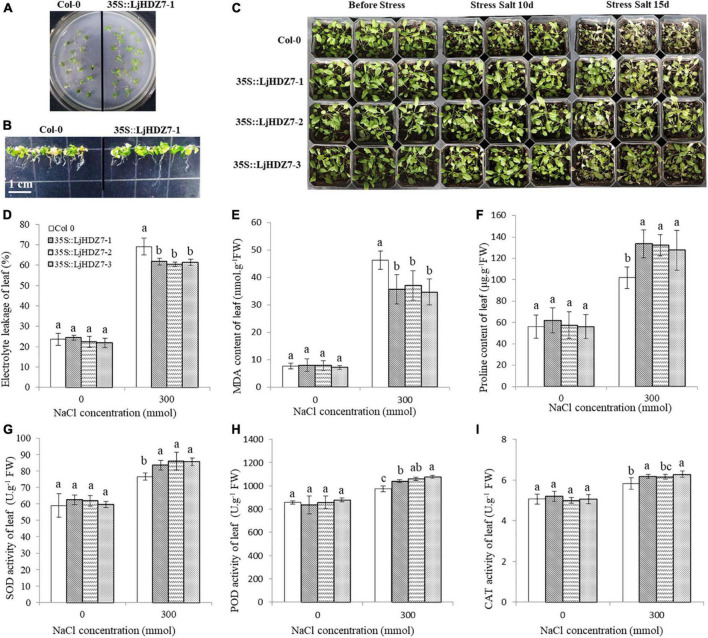
Overexpression of *LjHDZ7* in transgenic Arabidopsis improved salt tolerance. **(A)** Seed germination of wild-type (Col-0) and 35S:LjHDZ7-1 transgenic Arabidopsis plants in MS medium containing 150 mM NaCl. **(B)** Seeds of Col-0 and 35S:LjHDZ7-1 transgenic Arabidopsis were planted in MS medium for 7 days, and seedlings were transferred to MS medium containing 150 mM NaCl for 10 days before investigation. **(C)** Phenotypic analysis of Col-0 and 35S:LjHDZ7 transgenic Arabidopsis lines under salt tolerance. **(D)** Electrolyte leakage of leaf. **(E)** MDA content of leaf. **(F)** Proline content of leaf. **(G)** Superoxide dismutase (SOD) activity of leaf. **(H)** Peroxidase (POD) activity of leaf. **(I)** Catalase (CAT) activity of leaf. Data values are the means ± SD of three independent biological samples. Different letters indicate significance differences based on the LSD test (*p* < 0.05).

**FIGURE 5:**
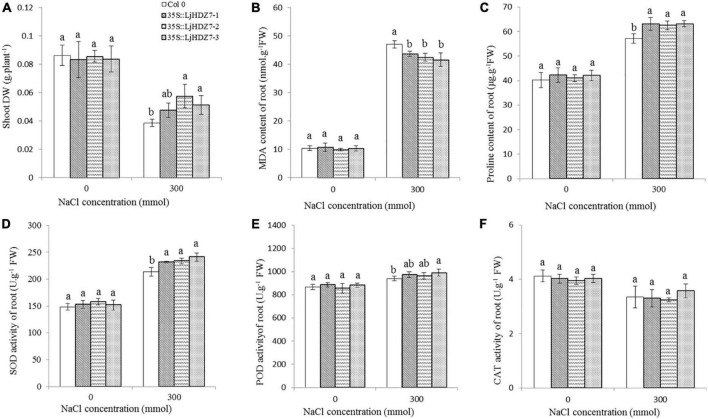
The effect of physiological index in Col-0 and *LjHDZ7* overexpression lines. **(A)** The shoot DW of Col-0 and *LjHDZ7* overexpression lines. **(B)** MDA content of root. **(C)** Proline content of root. **(D)** SOD activity of root. **(E)** POD activity of root. **(F)** CAT activity of root. Data values are the means ± SD of three independent biological samples. Different letters indicate significance differences based on the LSD test (*p* < 0.05).

Through sequence comparisons, we identified the homology between Lj0g3v0072079.1 and *AtHB7* (Supplementary Figure 14); therefore, we named the Lj0g3v0072079.1 gene *LjHDZ7* and subsequently overexpressed *LjHDZ7* in Arabidopsis. Our results showed that the seed germination of the *LjHDZ7* overexpression line was better than that of Col-0 under salt stress (Figure 4A). The root growth of the transgenic lines was greater than that of the Col-0 line under salt stress (Figure 4B). The leaves of Col-0 gradually turned yellow and died, whereas the leaves of the transgenic lines showed obviously better growth under salt stress (Figure 4C). The shoot drought weight (DW) of the transgenic lines was greater than that of the Col-0 line under salt stress (Figure 5A). The electrolyte leakage and MDA contents of Col-0 plants leaf were higher than those of the *LjHDZ7* overexpression lines (Figures 4D,E). The proline contents of the *LjHDZ7* overexpression lines leaf were higher than those of the Col-0 plants under salt stress (Figure 4F). Furthermore, the SOD, peroxidase (POD) and catalase (CAT) activities of the *LjHDZ7* overexpression lines leaf were significantly higher than those of the Col-0 plants under salt stress (Figures 4G–I). Similarly, MDA contents of Col-0 plants root was higher than that of the *LjHDZ7* overexpression lines, while proline contents and SOD activities of the *LjHDZ7* overexpression lines root were significantly higher than those of the Col-0 plants under salt stress (Figures 5B–F). These results showed that overexpressing *LjHDZ7* could increase the salt resistance of plants.

**FIGURE 6:**
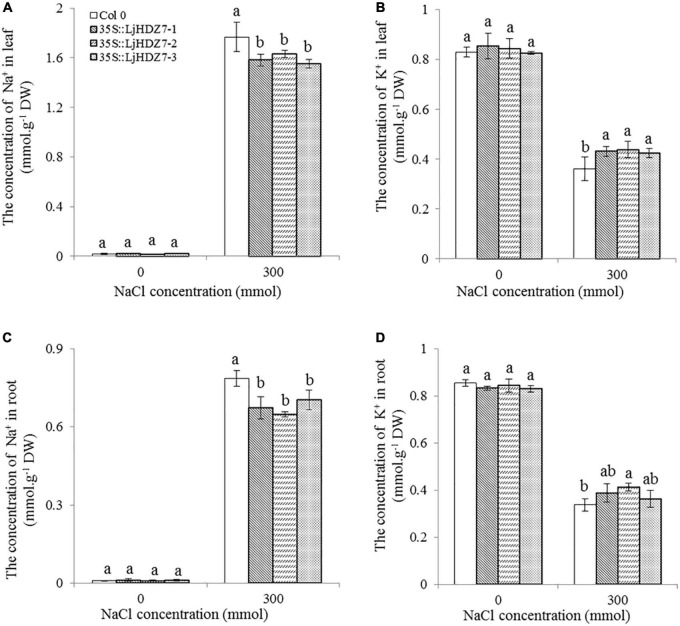
The concentrations of Na^+^ and K^+^ in the leaves and roots of Col-0 and *LjHDZ7*-overexpressing lines. The concentration of Na^+^ in leaves **(A)**, the concentration of K^+^ in leaves **(B)**, the concentration of Na^+^ in roots **(C)** and the concentration of K^+^ in roots **(D)** of Col-0 and *LjHDZ7* overexpression lines under control conditions and 300 mM NaCl. Data values are the means ± SD of three independent biological samples. Different letters indicate significant differences based on the LSD test (*p* < 0.05).

**FIGURE 7:**
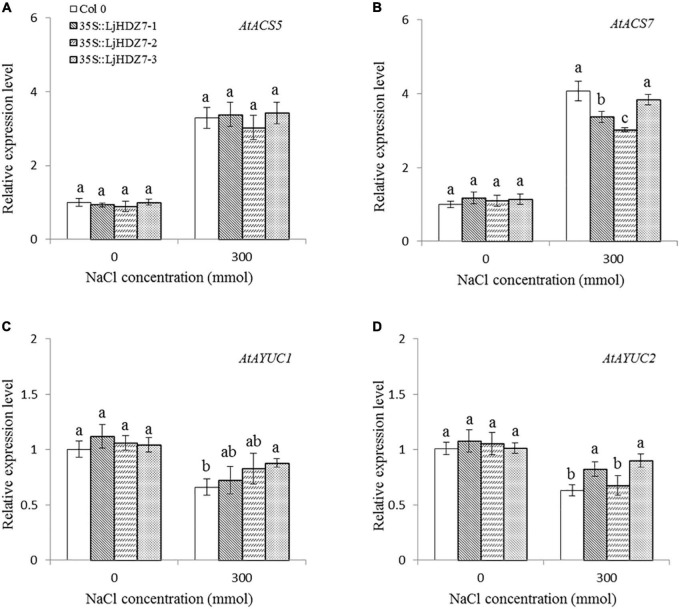
The expression levels of auxin and ethylene biosynthesis genes. The expression level of *AtACS5* **(A)**, the expression level of *AtACS7* **(B)**, the expression level of *AtYUC1* **(C)** and the expression level of *AtYUC2* **(D)** in Col-0 and *LjHDZ7* overexpression lines under control conditions and 300 mM NaCl. Data values are the means ± SD of three independent biological samples. Different letters indicate significant differences based on the LSD test (*p* < 0.05).

In this study, the concentrations of Na^+^ and K^+^ ions in leaves and roots were measured (Figure 6). Under 300 mM NaCl treatment, the concentration of Na^+^ ions in all plants significantly increased in the leaf and root parts, while the concentration of Na^+^ ions in *LjHDZ7* overexpression lines was significantly lower than that of Col-0 in both leaf and root parts (Figures 6A,C). Under 300 mM NaCl treatment, the concentration of K^+^ ions in all plants was significantly decreased in the leaf and root parts, but the concentration of K^+^ ions in *LjHDZ7* overexpression lines was significantly higher than that of Col-0 in the leaf parts (Figures 6B,D). Under 300 mM NaCl treatment, the expression of the ethylene biosynthesis genes *AtACS5* and *AtACS7* was obviously increased in Col-0 and transgenic plants, while the expression of *AtACS5* was not significantly different between Col-0 and the *LjHDZ7* overexpression lines. The expression of *AtACS7* in the two transgenic lines was significantly lower than that in Col-0 (Figures 7A,B). Under 300 mM NaCl treatment, the expression of the auxin biosynthesis genes *AtYUC1* and *AtYUC2* was slightly decreased in Col-0 and transgenic plants, but the expression of *AtYUC1* in *LjHDZ7* overexpression plants was slightly higher than that in Col-0, and one transgenic line reached the level of significance. The expression of *AtYUC2* in the two transgenic lines was significantly higher than that in Col-0 (Figures 7C,D).

## Discussion

Phylogenetic trees represent an established method for determining evolutionary changes and functional relationships (Li et al., 2015). The HD-Zip protein family has been identified in many species, from mosses to higher plants, such as *Ceratopteris richardii* (Aso et al., 1999), *Physcomitrium patens* (Yip et al., 2016), angiosperms and gymnosperms (Prigge and Clark, 2010). The phylogenetic analysis indicated that the HD-Zip gene family originated before the differentiation of vascular plants and moss lineages (Sakakibara et al., 2001). In our phylogenetic tree, a total of 186 HD-Zip proteins from three leguminous forage species and Arabidopsis were identified and divided into four subfamilies (Figure 1). This result was consistent with research reports on HD-Zip proteins in other species (Turchi et al., 2015). In addition, we also found that the three legumes had different numbers of HD-Zip genes and HD-Zip amino acid sequences (Supplementary Figure 1). Following species divergence and in conjunction with genome evolution, some members of the HD-Zip family diverged from their common ancestral genes (Jiang et al., 2015). Earlier findings showed that the exceptional increase in the sizes of TF families in higher plants is often related to WGD (whole-genome duplication) events (Lehti-Shiu et al., 2017). Legumes have undergone two WGDs within their MRCA (the Most Recent Common Ancestor). In particular, a WGD event resulted in major TF family expansions within the legumes (approximately 58 million years ago). Furthermore, TF family expansions occurred at the Glycine genus after a WGD (approximately 13 million years ago), which explains of the increase in most TF families in this genus (Moharana and Venancio, 2020). A study of legume genome evolution through the *Medicago truncatula* and *Lotus japonicus* genomes demonstrated WGD events early in legume evolution (Cannon et al., 2006). Therefore, the molecular evolution of the HD-Zip gene family in legumes shows that legume genomes have undergone extensive rearrangement, including translocation and inversion (Li et al., 2014).

Previous experiments have proven that the HD-Zip gene family has a variety of motifs and structures in plants that participate in various functions (Capella et al., 2014). Great differences were observed in the number and length of exons in each subgroup, suggesting that different gene structures indicated the different gene functions (Prigge et al., 2005). In our results, *Lotus japonicus* HD-Zip family proteins were all hydrophilic and localized to the nucleus (Supplementary Table 1), and they had different motifs, conserved domains, protein 3D structures (Supplementary Figures 3–5) and intron and exon numbers (Supplementary Figure 6). HD-Zip I and II were highly similar in protein structure, while HD-Zip III and IV had complex protein structures. Furthermore, HD-Zip proteins and the gene structure of each subfamily were very similar. The function of each subfamily was related to their structure. Each subfamily HD-Zip gene may have evolved from the same ancestor. HD-Zip I members have been proven to play critical roles in the regulation of plant developmental processes, signaling networks and responses to environmental stresses (Gong et al., 2019). HD-Zip I gene *LpHOX21* may act as a positive transcriptional regulator for heat tolerance in perennial ryegrass (Wang et al., 2019). Overexpression of the wheat *TaHDZipI-5* gene improved transgenic wheat freezing and drought resistance (Yang et al., 2017).

The response sequence of the gene promoter determines the specific expression pattern of genes and can reflect the function of genes (Butler and Kadonaga, 2002). In this study, a total of 95 *cis*-acting elements were detected in the promoter of *Lotus japonicus* HD-Zip genes (Supplementary Figure 8). We further found that 95 *cis*-acting elements contained stress-related elements (MBS, DRE, ERE, etc.) and hormone response elements (TCA-element, ABRE, TGA-element, et al.). Stress- and hormone-related elements are related to the stress function of genes (Zhou et al., 2021). All identified *cis*-elements were classified, and the stress and hormone response elements accounted for 10 and 16% in *Lotus japonicus* HD-Zip I, respectively (Figure 2A). The HD-Zip I gene promoters were classified into 14 types of stress-related *cis*-elements, including ABA- and salt stress-related *cis*-elements (Figure 2B). Therefore, the *Lotus japonicus* HD-Zip I gene promoter could respond to stress factors and HD-Zip I members could play an important role in plant abiotic stress.

Transcription factor can regulate plant responses to hormones and environmental factors by regulating gene expression (Nath et al., 2019). Studies have shown that the expression of HD-Zip subfamily I TFs is induced by drought, high salt, ABA and chilling injury (Manavella et al., 2008). Here, our results showed that the expression of six selected HD-Zip I members was induced by different stresses (Figure 3D). These six genes had differential expression abundance in different stress types and stress time points; therefore, they may be involved in different adversity pathways. Expression of the HD-Zip family *HaHB11* was induced by ABA, NaCl and water deficit in sunflower (Cabello et al., 2016). Arabidopsis *AtHB13* was upregulated by low temperatures and played a key role in cold, drought, and salinity stresses (Cabello and Chan, 2012; Cabello et al., 2015). We further found that the expression of the *LjHDZ7* gene was strongly induced by abiotic stress, especially salt stress (Figure 3B). Therefore, *LjHDZ7* may play an important role in salt stress.

Homeodomain-leucine zipper genes participate in the hormone signaling pathway and regulate plant cell expansion, division and differentiation by interacting with hormone pathway genes and downstream genes to improve plant stress tolerance (Agalou et al., 2008). In our study, many proteins related to the stress response were predicted to interact with LjHDZ7, such as RD26 (Fujita et al., 2004) and WRKY (Singh et al., 2017), ABA receptor protein, ABA signaling ABI and HAB (Supplementary Figure 11A). Furthermore, the homologous family of PP2C was identified and showed different expression levels under different stressors in *Lotus japonicus* (Supplementary Figure 11B). PYL and PP2C are key factors in the ABA pathway (Cheng et al., 2017). As previously reported for HD-Zip proteins in *Arabidopsis thaliana, AtHB12* is involved in ABA signaling by regulating PP2C and ABA receptor gene activities (Valdés et al., 2012). Therefore, our results indicated that *LjHDZ7* may regulate the stress response by the ABA signaling pathway, which needs to be further investigated.

The combination of modern molecular biology and traditional breeding methods can improve breeding efficiency. A drought-induced HD-Zip I gene, *Zmhdz10*, was isolated from maize and positively regulated drought and salt tolerance in both genetically transformed rice and Arabidopsis (Zhao et al., 2014). Overexpression of the wheat *TaHDZipI-5* gene increased transgenic wheat freezing and drought resistance (Yang et al., 2017). We cloned the *LjHDZ7* gene and obtained *LjHDZ7*-overexpressing transgenic Arabidopsis plants. Under salt stress, the growth of the transgenic lines was obviously better than that of the Col-0 line (Figures 4A–C). The electrolyte leakage and MDA contents of Col-0 plants were higher than those of the *LjHDZ7* overexpression lines (Figures 4D,E, 5B,C). Furthermore, the proline contents, SOD, POD, and CAT activities of *LjHDZ7* overexpression lines were higher than those of Col-0 plants (Figures 4F–I, 5D–F). These results showed that the overexpression of *LjHDZ7* could increase the salt resistance of plants. Overexpression of *PsnHDZ63* resulted in improved reactive oxygen species scavenging ability and enhanced salt stress tolerance in transgenic *Populus simonii* × *P. nigra* (Guo et al., 2021). HD-Zip I gene *BpHOX2* could bind to different cis-acting elements to regulate gene expression, thus improving osmotic tolerance in birch (Tan et al., 2020). Therefore, the HD-Zip gene family may become valuable to improve stress tolerance in molecular breeding methods.

Excessive Na^+^ in plants could result in ion toxicity, which causes an imbalance in ion uptake and nutritional disorders and finally leads to growth inhibition and even plant death (Wu et al., 2019; Zhang H. et al., 2020). Maintaining K^+^ and Na^+^ ion homeostasis is important for a series of plant physiological and biochemical processes and for resistance to salinity (Ma et al., 2017). In the present study, the concentration of Na^+^ ions in *LjHDZ7* overexpression lines was significantly lower than that in Col-0 in both leaf and root parts (Figures 6A,C). Furthermore, the concentration of K^+^ ions in *LjHDZ7* overexpression lines was significantly higher than that in Col-0 in the leaf parts (Figures 6B,D). These results indicated that *LjHDZ7* overexpression lines may have a stronger selective absorption and transportation capacity for K^+^ and Na^+^ and could maintain a higher concentration of K^+^ under saline conditions.

The phytohormone ethylene plays essential roles in plant abiotic stress adaptation. ACS (ACC synthase) is the key enzyme in ethylene biosynthesis. A previous study showed that the expression of *AtACS7* was regulated by salt stress (Dong et al., 2011). In this study, under 300 mM NaCl treatment, the expression of the ethylene biosynthesis genes *AtACS5* and *AtACS7* was obviously increased in both Col-0 and *LjHDZ7* overexpression lines, while there was no significant difference between Col-0 and *LjHDZ7* overexpression lines (Figures 7A,B). There may be differences in the expression of other *AtACS* genes between Col-0 and *LjHDZ7* overexpression lines. Auxin regulates many of the key processes in plant growth and stress responses. The YUCCA (YUC) family is the key limiting enzyme in the auxin biosynthetic pathway (Liu et al., 2015). A previous study showed that plant IAA levels were significantly decreased under salt stress (Hofmann, 2011). Our study showed that the expression of the *AtYUC1* and *AtYUC2* genes was downregulated in Col-0 and *LjHDZ7* overexpression plants under salt treatment. The expression of *AtYUC2* in two transgenic lines was significantly higher than that in Col-0 plants (Figures 7C,D). These results showed that *LjHDZ7* could change endogenous ethylene and auxin levels by regulating the expression of ethylene and auxin biosynthesis genes under salt treatment.

## Conclusion

We performed an evolutionary analysis of the HD-Zip family in three leguminous forage species to obtain comprehensive information on this family, including the protein features and phylogenetic relationships. Furthermore, the motifs and domains of the proteins, gene structures, promoter *cis*-elements and stress expression patterns of HD-Zip genes were analyzed in *Lotus japonicus*. We further found that the expression of the *LjHDZ7* gene was strongly induced by abiotic stress, especially salt stress. Furthermore, our results showed that overexpression of *LjHDZ7* could increase salt resistance in transgenic Arabidopsis plants. Our results are important because they provide a theoretical basis for further functional studies of the HD-Zip gene family in *Lotus japonicus* and resources for prospective applications for stress-resistant breeding.

## Data availability statement

The datasets presented in this study can be found in online repositories. The names of the repository/repositories and accession number(s) can be found in the article/Supplementary material.

## Author contributions

DW and SN wrote the main manuscript text and contributed to the conception of the study. DW, YG, YL, and SN performed the experiments and the data analyses. All authors have read and agreed to the published version of the manuscript.

## Footnotes

1
http://planttfdb.gao-lab.org/

2
http://www.ncbi.nlm.nih.gov/Structure/cdd/wrpsb.cgi

3
http://smart.embl-heidelberg.de/smart/set_mode.cgi?NORMAL=1

4
http://web.expasy.org/protparam/

5
https://www.uniprot.org/

6
https://wolfpsort.hgc.jp/

7
http://meme-suite.org/

8
http://www.sbg.bio.ic.ac.uk/∼phyre2/html/page.cgi?id=index

9
http://www.kazusa.or.jp/lotus/

10
http://gsds.cbi.pku.edu.cn/

11
http://mg2c.iask.in/mg2c_v2.1/

12
http://bioinformatics.psb.ugent.be/webtools/plantcare/html/

13
https://string-db.org/cgi/input.pl

## Supplementary material

Supplementary Figure S1 Statistical analysis of the HD-Zip subfamily in each plant species. **(A)** Number of HD-Zip genes found in each subfamily of *Lotus japonicus*, *Medicago truncatula*, *Trifolium pratense*, and *Arabidopsis thaliana*. **(B–E)** Amino acid sequences of HD-Zip genes in *Arabidopsis thaliana*, *Medicago truncatula*, *Trifolium pretense*, and *Lotus japonicus*, respectively. Genes corresponding to **(B–E)** X-axis NO were in Supplementary Tables S1–S4, respectively.

Supplementary Figure S2 Distribution of HD-Zip subfamilies within four plant species.

Supplementary Figure S3 Conserved motif and domain analysis of HD-Zip proteins from *Lotus japonicus.* The phylogenetic tree was generated based on the protein sequences of HD-Zip proteins. Different colored boxes represent different types of motifs and domains.

Supplementary Figure S4 Sequence information for Motif 1- Motif 10.

Supplementary Figure S5 Three-dimensional modeling structure of HD-Zip proteins in *Lotus japonicus*.

Supplementary Figure S6 **(A)** Chromosome localization of HD-Zip in *Lotus japonicus*. Location of HD-Zip genes in *Lotus japonicus*. **(B)** Number of four distinct HD-Zip subfamilies per chromosome.

Supplementary Figure S7 Gene structure of the HD-Zip family of *Lotus japonicus.* **(A)** Exon/intron structures of HD-Zip genes of *Lotus japonicus*. Exons and introns are represented by yellow bones and gray lines, respectively. **(B)** Exon and gene numbers of four HD-Zip subfamilies of *Lotus japonicus*.

Supplementary Figure S8 *Cis*-element identification in the promoters of HD-Zip from *Lotus japonicus.*

Supplementary Figure S9 Promoters *cis*-element analysis of HD-Zip of *Lotus japonicus*. *Cis*-element statistical analysis of **(A)** HD-Zip II, **(B)** HD-Zip III, and **(C)** HD-Zip IV family gene promoters related to stress and hormone responses. Different *cis*-elements with similar functions are shown in the same color.

Supplementary Figure S10 Expression levels of nine HD-Zip II, III and HD-Zip IV genes under various stresses in different times. **(A)** Expression level of Lj4g3v2140210.1 during different stresses and times. **(B)** Lj2g3v1989250.1. **(C)** Lj3g3v0463690.1. **(D)** Lj4g3v2665270.1. **(E)** Lj3g3v1074990.2. **(F)** Lj0g3v0278949.1. **(G)** Lj1g3v2611340.1. **(H)** Lj0g3v0251169.1. **(I)** Lj0g3v0262429.2.

Supplementary Figure S11 Predicted protein interaction networks of Lj0g3v0072079.1. **(A)** Protein interaction network of Lj0g3v0072079.1 was generated using the STRING online database, with *Arabidopsis thaliana* as the background. Edges represent protein–protein associations. The nodes represent proteins, and red nodes represent query proteins. The black, green, blue, light sky blue and purple lines represent co-expression, text mining, gene co-occurrence, protein homology, and experimental determination, respectively. **(B)** Relative expression levels of Lj4g3v3044980.2 and Lj6g3v1211810.1 under different stresses at 24 h.

Supplementary Figure S12 Predicted protein interaction networks of Lj0g3v0072079.1, with *Medicago truncatula* as the background.

Supplementary Figure S13 Amino acid sequence alignment of the protein phosphatase 2C (PP2C) family. Including Lj4g3v3044980.2 and Lj6g3v1211810.1 from *Lotus japonicus* and ATABI1, ATABI2, ATHAB1, and ATHAB2 from *Arabidopsis thaliana*.

Supplementary Figure S14 Amino acid sequence alignment of LjHDZ7, ATHB-7, and AET01987.

Supplementary Table S2 Basic physicochemical properties of the HD-Zip family in *Arabidopsis thaliana*.

Supplementary Table S3 Basic physicochemical properties of the HD-Zip family in *Medicago truncatula*.

Supplementary Table S4 Basic physicochemical properties of the HD-Zip family in *Trifolium pratense*.

## Abbreviations

HD-Zip: the homeodomain leucine zipper
TF: transcription factor
Mw: molecular weight
pI: isoelectric point
SA: salicylic acid
JA: jasmonic acid
MeJA: methyl jasmonate
GA: gibberellin
q-PCR: quantitative polymerase chain reaction
ABA: abscisic acid
MDA: malonaldehyde
PEG6000: polyethylene glycol 6000
NCBI: National Center for Biotechnology Information.
