# Supplementary material for: Genome-wide analysis of the homeodomain-leucine zipper family in Lotus japonicus and the overexpression of LjHDZ7 in Arabidopsis for salt tolerance
Source: Front Plant Sci. 2022 Sep 14;13:955199. doi: 10.3389/fpls.2022.955199 (PMC9515785; doi:10.3389/fpls.2022.955199)
Supplement: Supplementary file 1 [file Table_1.DOCX]

Table S1 Protein physical and chemical properties of HD-ZIP family in *Lotus japonicus*

| Group  Name | NO. | ID Number | AA | MW  (Da) | pI | GRAVY | AtHDZ Ortholog | Subcellular localization |
| --- | --- | --- | --- | --- | --- | --- | --- | --- |
| Ⅰ | 1 | Lj2g3v1349130.1 | 320 | 36246.8 | 4.62 | -0.921 | AT3G01470.1 | Nucleus |
| Ⅰ | 2 | Lj4g3v1614670.1 | 318 | 35788.5 | 4.67 | -0.744 | AT3G01470.1 | Nucleus |
| Ⅰ | 3 | Lj2g3v1014380.1 | 167 | 19798.1 | 5.81 | -1.123 | AT3G01470.1 | Nucleus |
| Ⅰ | 4 | Lj0g3v0279669.1 | 207 | 24088.2 | 6.92 | -0.992 | AT5G03790.1 | Nucleus |
| Ⅰ | 5 | Lj1g3v4693000.1 | 182 | 21655.4 | 7.72 | -1.129 | AT5G03790.1 | Nucleus |
| Ⅰ | 6 | Lj0g3v0266959.1 | 266 | 30603.4 | 8.64 | -0.882 | AT3G01220.1 | Nucleus |
| Ⅰ | 7 | Lj2g3v1034880.1 | 305 | 34691.7 | 6.36 | -0.849 | AT3G01220.1 | Nucleus |
| Ⅰ | 8 | Lj2g3v1316330.1 | 201 | 23244.1 | 7.82 | -0.965 | AT1G69780.1 | Nucleus |
| Ⅰ | 9 | Lj2g3v1327450.1 | 280 | 32052.6 | 6.16 | -0.998 | AT1G69780.1 | Nucleus |
| Ⅰ | 10 | Lj0g3v0168929.1 | 329 | 37231.0 | 4.89 | -0.887 | AT4G40060.1 | Nucleus |
| Ⅰ | 11 | Lj6g3v1053520.1 | 257 | 30027.6 | 5.85 | -0.939 | AT5G65310.1 | Nucleus |
| Ⅰ | 12 | Lj0g3v0072079.1 | 246 | 28145.2 | 5.07 | -0.944 | AT2G46680.1 | Nucleus |
| Ⅰ | 13 | Lj1g3v1037350.1 | 240 | 28008.0 | 5.24 | -1.186 | AT2G46680.2 | Nucleus |
| Ⅱ | 14 | Lj0g3v0103139.1 | 266 | 29854.9 | 8.35 | -0.752 | AT4G37790.1 | Nucleus |
| Ⅱ | 15 | Lj2g3v1984020.1 | 267 | 29872.9 | 8.72 | -0.712 | AT2G22800.1 | Nucleus |
| Ⅱ | 16 | Lj1g3v1782140.1 | 288 | 32664.0 | 8.81 | -0.832 | AT4G37790.1 | Nucleus |
| Ⅱ | 17 | Lj4g3v2140210.1 | 205 | 23735.2 | 9.04 | -0.925 | AT4G37790.1 | Nucleus |
| Ⅱ | 18 | Lj6g3v1300850.1 | 232 | 26537.8 | 6.80 | -1.012 | AT4G37790.1 | Nucleus |
| Ⅱ | 19 | Lj2g3v1989250.1 | 304 | 33856.9 | 6.68 | -0.799 | AT4G16780.1 | Nucleus |
| Ⅱ | 20 | Lj4g3v1218330.1 | 233 | 26378.7 | 7.08 | -0.814 | AT4G16780.1 | Nucleus |
| Ⅱ | 21 | Lj3g3v0515110.1 | 311 | 34245.1 | 8.45 | -0.905 | AT3G60390.1 | Nucleus |
| Ⅱ | 22 | Lj1g3v4350060.1 | 333 | 36907.4 | 7.59 | -0.724 | AT5G06710.1 | Nucleus |
| Ⅱ | 23 | Lj3g3v0927080.1 | 358 | 38961.8 | 6.17 | -0.914 | AT5G06710.1 | Nucleus |
| Ⅱ | 24 | Lj4g3v0633410.1 | 117 | 13214.4 | 9.80 | -0.492 | AT2G01430.1 | Nucleus |
| Ⅱ | 25 | Lj3g3v0463690.1 | 234 | 26797.3 | 9.25 | -0.881 | AT5G06710.1 | Nucleus |
| Ⅲ | 26 | Lj4g3v2665270.1 | 619 | 68042.8 | 6.38 | -0.174 | AT1G30490.1 | Nucleus |
| Ⅲ | 27 | Lj6g3v1654310.1 | 488 | 53947.4 | 7.53 | -0.320 | AT2G34710.1 | Nucleus |
| Ⅲ | 28 | Lj3g3v1074990.2 | 841 | 92039.8 | 5.96 | -0.093 | AT5G60690.1 | Nucleus |
| Ⅲ | 29 | Lj3g3v3085530.1 | 843 | 92512.4 | 5.96 | -0.116 | AT5G60690.1 | Nucleus |
| Ⅲ | 30 | Lj0g3v0278949.1 | 304 | 33880.0 | 8.60 | -0.260 | AT1G52150.3 | Nucleus |
| Ⅳ | 31 | Lj2g3v1509550.1 | 776 | 86687.5 | 5.56 | -0.381 | AT5G46880.1 | Nucleus |
| Ⅳ | 32 | Lj3g3v0247270.1 | 641 | 71535.7 | 6.84 | -0.316 | AT1G79840.1 | Nucleus |
| Ⅳ | 33 | Lj0g3v0251169.1 | 738 | 80983.1 | 6.01 | -0.434 | AT4G00730.1 | Nucleus |
| Ⅳ | 34 | Lj5g3v2017090.1 | 661 | 73510.8 | 5.98 | -0.414 | AT3G61150.1 | Nucleus |
| Ⅳ | 35 | Lj0g3v0360389.1 | 618 | 68344.6 | 6.53 | -0.463 | AT4G00730.1 | Nucleus |
| Ⅳ | 36 | Lj1g3v2611340.1 | 810 | 88258.0 | 5.88 | -0.307 | AT4G00730.1 | Nucleus |
| Ⅳ | 37 | Lj0g3v0262429.2 | 535 | 59396.1 | 5.98 | -0.482 | AT1G05230.4 | Nucleus |
| Ⅳ | 38 | Lj3g3v1338000.1 | 740 | 80630.1 | 5.69 | -0.313 | AT1G05230.4 | Nucleus |
| Ⅳ | 39 | Lj1g3v0052560.1 | 728 | 79980.5 | 5.91 | -0.325 | AT4G21750.2 | Nucleus |
| Ⅳ | 40 | Lj3g3v1541360.1 | 728 | 79980.5 | 5.91 | -0.325 | AT4G21750.2 | Nucleus |

AA, Number of amino acids; MW, Molecular weight; pI, Theoretical pI; GRAVY, Grand average of hydropathicity.
