# Supplementary material for: Genome-wide analysis of the homeodomain-leucine zipper family in Lotus japonicus and the overexpression of LjHDZ7 in Arabidopsis for salt tolerance
Source: Front Plant Sci. 2022 Sep 14;13:955199. doi: 10.3389/fpls.2022.955199 (PMC9515785; doi:10.3389/fpls.2022.955199)
Supplement: Supplementary file 2 [file Table_5.DOCX]

**Table S5. Predicted subcellular localization of HD-ZIP proteins in *Lotus japonicus***

| ID Number | WoLF PSORT Prediction |
| --- | --- |
| Lj2g3v1014380.1 | nucl: 14 |
| Lj6g3v1053520.1 | nucl: 14 |
| Lj0g3v0072079.1 | nucl: 14 |
| Lj0g3v0168929.1 | nucl: 14 |
| Lj0g3v0266959.1 | nucl: 14 |
| Lj0g3v0279669.1 | nucl: 14 |
| Lj1g3v1037350.1 | nucl: 14 |
| Lj1g3v4693000.1 | nucl: 14 |
| Lj2g3v1034880.1 | nucl: 14 |
| Lj2g3v1316330.1 | nucl: 14 |
| Lj2g3v1327450.1 | nucl: 13.5, cyto_nucl: 7.5 |
| Lj2g3v1349130.1 | nucl: 12.5, cyto_nucl: 7, pero: 1 |
| Lj4g3v1614670.1 | nucl: 14 |
| Lj1g3v1782140.1 | nucl: 14 |
| Lj0g3v0103139.1 | nucl: 13, pero: 1 |
| Lj1g3v4350060.1 | nucl: 13, pero: 1 |
| Lj6g3v1300850.1 | nucl: 13, pero: 1 |
| Lj2g3v1984020.1 | nucl: 14 |
| Lj2g3v1989250.1 | nucl: 13, pero: 1 |
| Lj3g3v0463690.1 | chlo: 9, nucl: 4, plas: 1 |
| Lj3g3v0515110.1 | nucl: 13, pero: 1 |
| Lj3g3v0927080.1 | nucl: 14 |
| Lj4g3v0633410.1 | chlo: 13, nucl: 1 |
| Lj4g3v1218330.1 | nucl: 13, pero: 1 |
| Lj4g3v2140210.1 | nucl: 14 |
| Lj0g3v0278949.1 | golg: 4, cyto: 3, E.R.: 3, nucl: 2, chlo: 1, vacu: 1 |
| Lj6g3v1654310.1 | nucl: 5, chlo: 4, cyto: 4, cysk: 1 |
| Lj4g3v2665270.1 | chlo: 6, cyto: 4, nucl: 3, cysk: 1 |
| Lj3g3v3085530.1 | chlo: 8, nucl: 3, cyto: 1, vacu: 1, cysk: 1 |
| Lj3g3v1074990.2 | cyto: 6, nucl: 5, chlo: 2, vacu: 1 |
| Lj3g3v1338000.1 | nucl: 12, extr: 2 |
| Lj1g3v2611340.1 | nucl: 13, pero: 1 |
| Lj2g3v1509550.1 | nucl: 14 |
| Lj3g3v0247270.1 | nucl: 13, pero: 1 |
| Lj3g3v1541360.1 | nucl: 14 |
| Lj5g3v2017090.1 | nucl: 14 |
| Lj0g3v0251169.1 | nucl: 14 |
| Lj0g3v0262429.2 | nucl: 14 |
| Lj1g3v0052560.1 | nucl: 14 |
| Lj0g3v0360389.1 | nucl: 14 |

nucl, nuclus; cyto, cytoplasimic; pero: peroxisome; chlo, choloplast; plas, plasma membrane; golg: golgi bodies; vacu: vacuole; cysk, cytoskeleton. The numbers indicate. 14 Nearest Neighbors.
