# Supplementary material for: Genome-wide analysis of the homeodomain-leucine zipper family in Lotus japonicus and the overexpression of LjHDZ7 in Arabidopsis for salt tolerance
Source: Front Plant Sci. 2022 Sep 14;13:955199. doi: 10.3389/fpls.2022.955199 (PMC9515785; doi:10.3389/fpls.2022.955199)
Supplement: Supplementary file 3 [file Table_6.DOCX]

**Table S6. Major MEME motif sequence in *Lotus japonicus* HD-Zip protein**

| Motif | Width | Best possible match |
| --- | --- | --- |
| 1 | 29 | ZLGLQPRQVKVWFQNRRARWKTKQEEVDY |
| 2 | 29 | RKKLRLTKEQVQALERSFKECPKLNPKQK |
| 3 | 50 | LPSGCVIQDMPNGYSKITWVEHVEVDERSVHZLYRPLVESGKAFGAKRWI |
| 4 | 42 | STGVPGNNNGAJZLMYAELQAPTPLVPTREFYFLRYCKQHED |
| 5 | 21 | NENLTEENARLQKEVZELKAL |
| 6 | 50 | TSVWLPVSPKRVFDFLRDENSRSZWDILSNGGPVQEMAHIAKGQDPGNCV |
| 7 | 49 | KPSGFRSEASRETGVVIMNSVALVETLMDANQWAEMFPCIVSRAATLEV |
| 8 | 35 | JLRQENEKLRAENMRLREALSNPSCPNCGGPAMIG |
| 9 | 29 | SADGRKSMLKLAZRMTNNFCAGVCASSAH |
| 10 | 29 | ADKPIJLEJAVAAMEELIKMAQTGEPLWV |
