# Supplementary material for: Genome-wide analysis of the homeodomain-leucine zipper family in Lotus japonicus and the overexpression of LjHDZ7 in Arabidopsis for salt tolerance
Source: Front Plant Sci. 2022 Sep 14;13:955199. doi: 10.3389/fpls.2022.955199 (PMC9515785; doi:10.3389/fpls.2022.955199)
Supplement: Supplementary file 4 [file Table_7.DOC]

Table S7. Primers used in the present study

| Primer | Sequence (5' to 3') |
| --- | --- |
| LjUbi-F | CAAGGAAGGTATCCCACCG |
| LjUbi-R | TTAGAATCCACCACGAAGACG |
| Lj0g3v0266959.1-F | CTTTTCCTCTTTGCCACCCC |
| Lj0g3v0266959.1-R | CTGCCTCAAGTTTGTTCCCC |
| Lj2g3v1034880.1-F | TCAACCTCTCTCAACGCCTT |
| Lj2g3v1034880.1-R | GACAAATCATCGTCTCCGCC |
| Lj2g3v1316330.1-F | GCACTTCAAGCTCACAACCA |
| Lj2g3v1316330.1-R | AGCAGAGGGTGGAAAGAGTG |
| Lj2g3v1327450.1-F | GCACTTCAAGCTCACAACCA |
| Lj2g3v1327450.1-R | AGCAGAGGGTGGAAAGAGTG |
| Lj0g3v0072079.1-F | ACACCACTAGCAGCTCAACT |
| Lj0g3v0072079.1-R | CAACCTGTCTTGGCTGCAAT |
| Lj1g3v1037350.1-F | GGGTTCAGGAAGTGGGTACA |
| Lj1g3v1037350.1-R | TGCCTGGTCTAAGATCCCAC |
| Lj4g3v3044980.2-F | AGGGAGTAAGCTGGGGACAT |
| Lj4g3v3044980.2-R | CCGAAGAAATGAATGGGAGA |
| Lj6g3v1211810.1-F | CTTGCAAGAGCAATGGAACA |
| Lj6g3v1211810.1-R | TTGAGTCAAAATGGCAACCA |
| Lj4g3v2140210.1-F  Lj4g3v2140210.1-R | TGCTTCAAGCTGCATACCAC  CTCCAACTTTCAATGCACGA |
| Lj2g3v1989250.1-F | AGGAAGCAGGGGTTTCATCT |
| Lj2g3v1989250.1-R | GCCAGTGCCATCTTTTGTTT |
| Lj3g3v0463690.1-F | TTCAAGGCAACCTCCAATTC |
| Lj3g3v0463690.1-R | AGGCTCTTCCATGAAGCTGA |
| Lj4g3v2665270.1-F | GCCTTTGGCTGGTGATATGT |
| Lj4g3v2665270.1-R | AATGCTATGATGCGGAAACC |
| Lj3g3v1074990.2-F | CATGGCACGTCAGTATGTCC |
| Lj3g3v1074990.2-R | GCAGTCCAGTCCCCAAATAA |
| Lj0g3v0278949.1-F | CGCCAACAACTCATCAGAGA |
| Lj0g3v0278949.1-R | TGACACCTGCTTCTGCAATC |
| Lj0g3v0251169.1-F | GTATCGCGACAAAGGGTGTT |
| Lj0g3v0251169.1-R | CTGAACGTCGATGGAGGAAT |
| Lj1g3v2611340.1-F | GGCTTGAACTCAGCAAAAGG |
| Lj1g3v2611340.1-R | AGGACCACCACAATTTGAGC |
| Lj0g3v0262429.2-F | CTTGGATAATTTGCGCCCTA |
| AtACS5-F | CGGCAAGTCTCAAGAGGAAC |
| AtACS5-R | TCTGCAAGGCAAAACATGAG |
| AtACS7-F | TGCTGGCTTCTATGTTGTCG |
| AtACS7-R | ATCACATCCCAAAGCTGGAG |
| AtYUC1-F | GAACACCGTTCATGTGTTGC |
| AtYUC1-R | GACTTTTGCCGGTGACATTT |
| AtYUC2-F | GAGCCTGCTCAAGTGGTTTC |
| AtYUC2-R | CCAACGTCCAAAACAGGAGT |
