# Supplementary material for: Genome-wide analysis of the homeodomain-leucine zipper family in Lotus japonicus and the overexpression of LjHDZ7 in Arabidopsis for salt tolerance
Source: Front Plant Sci. 2022 Sep 14;13:955199. doi: 10.3389/fpls.2022.955199 (PMC9515785; doi:10.3389/fpls.2022.955199)
Supplement: Supplementary file 5 [file Table_8.docx]

**Table S8. Functional enrichments in the network of Lj0g3v0072079.1 proteins**

| Gene Ontology | cluster | | Description |
| --- | --- | --- | --- |
| Biological Process | GO:0009788 | Negative regulation of abscisic acid-activated signaling pathway | |
|  | GO:0009738 | Abscisic acid-activated signaling pathway | |
|  | GO:0009414 | Response to water deprivation | |
|  | GO:0048585 | Negative regulation of response to stimulus | |
|  | GO:0009737 | Response to abscisic acid | |
| Molecular Function | GO:0004724 | Magnesium-dependent protein serine/threonine phosphatase activity | |
|  | GO:0043565 | Sequence-specific dna binding | |
|  | GO:0003700 | DNA-binding transcription factor activity | |
|  | GO:0003677 | DNA binding | |
|  | GO:0003676 | Nucleic acid binding | |
|  | GO:0005488 | Binding | |
| Cellular Component | GO:0005634 | Nucleus | |

Functional enrichments in the network originate from Gene Ontology.
