# Supplementary figures and images for: Genome-wide analysis of the homeodomain-leucine zipper family in Lotus japonicus and the overexpression of LjHDZ7 in Arabidopsis for salt tolerance
Source: Front Plant Sci. 2022 Sep 14;13:955199. doi: 10.3389/fpls.2022.955199 (PMC9515785; doi:10.3389/fpls.2022.955199)

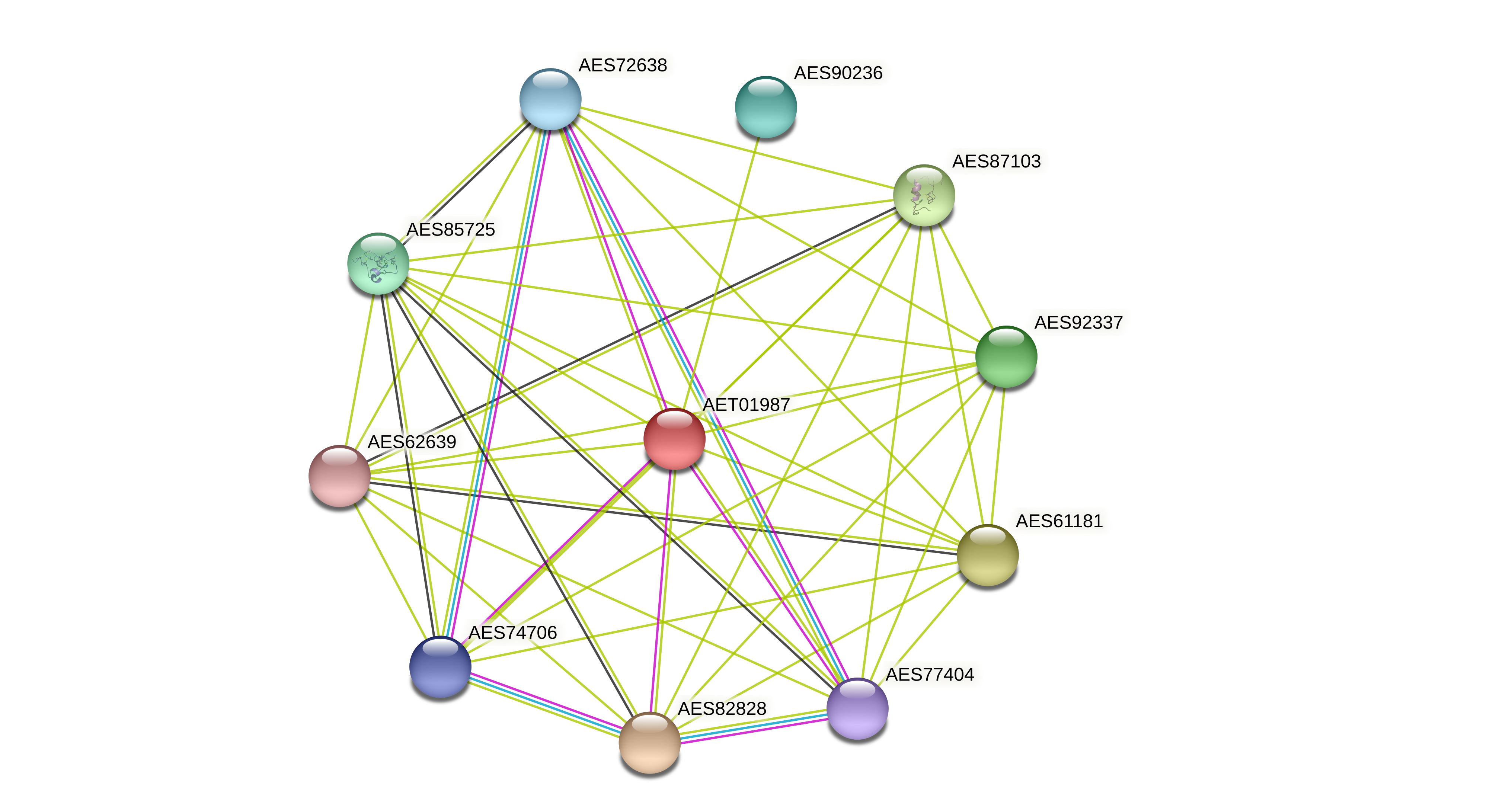

Supplement: Supplementary Figure S12 — Predicted protein interaction networks of Lj0g3v0072079.1, with Medicago truncatula as the background. [file Image_12.PNG]
